# Impairment of cerebral autoregulation in pediatric extracorporeal membrane oxygenation associated with neuroimaging abnormalities

**DOI:** 10.1117/1.NPh.4.4.041410

**Published:** 2017-08-19

**Authors:** Fenghua Tian, Michael Craig Morriss, Lina Chalak, Ramgopal Venkataraman, Chul Ahn, Hanli Liu, Lakshmi Raman

**Affiliations:** a University of Texas at Arlington, Department of Bioengineering, Arlington, Texas, United States; b University of Texas Southwestern Medical Center, Department of Radiology, Dallas, Texas, United States; c University of Texas Southwestern Medical Center, Department of Pediatrics, Dallas, Texas, United States; d University of Texas at Arlington, Department of Accounting, Arlington, Texas, United States; e University of Texas Southwestern Medical Center, Department of Clinical Science, Dallas, Texas, United States

**Keywords:** extracorporeal membrane oxygenation, cerebral autoregulation, wavelet transform coherence, neurological injury, blood pressure, cerebral tissue oxygen saturation

## Abstract

Extracorporeal membrane oxygenation (ECMO) is a life-supporting therapy for critically ill patients with severe respiratory and/or cardiovascular failure. Cerebrovascular impairment can result in hemorrhagic and ischemic complications commonly seen in the patients supported on ECMO. We investigated the degree of cerebral autoregulation impairment during ECMO as well as whether it is predictive of neuroimaging abnormalities. Spontaneous fluctuations of mean arterial pressure (MAP) and cerebral tissue oxygen saturation ($S_{\mathrm{ct}}O_{2}$) were continuously measured during the ECMO run. The dynamic relationship between the MAP and $S_{\mathrm{ct}}O_{2}$ fluctuations was assessed based on wavelet transform coherence (WTC). Neuroimaging was conducted during and/or after ECMO as standard of care, and the abnormalities were evaluated based on a scoring system that had been previously validated among ECMO patients. Of the 25 patients, 8 (32%) had normal neuroimaging, 7 (28%) had mild to moderate neuroimaging abnormalities, and the other 10 (40%) had severe neuroimaging abnormalities. The degrees of cerebral autoregulation impairment quantified based on WTC showed significant correlations with the neuroimaging scores (R=0.66; p<0.0001). Evidence that cerebral autoregulation impairment during ECMO was related to the patients’ neurological outcomes was provided.

## 1 Introduction

Extracorporeal membrane oxygenation (ECMO)^1^ is a life-supporting therapy for critically ill patients with severe respiratory and/or cardiovascular failure. It is also used as part of cardiopulmonary resuscitation (CPR) when conventional CPR fails. The use of ECMO support has increased steadily since the early 1990s with improved survival rates, averaging 80% in neonates and 65% in children.^2^ This decreased mortality has unmasked neurological injury that may be subclinical during ECMO but is an important cause of morbidity, leading to long-term neurocognitive complications.^3^^,^^4^

The healthy brain is protected by cerebral autoregulation, which maintains an adequate cerebral blood flow (CBF) in face of blood pressure changes.^5^ Pre-ECMO factors, such as hypoxia, hypercarbia, and hypertension, can disrupt blood flow regulation, leaving the brain vulnerable to changes in blood pressure.^6^ Cannulation of great blood vessels^7^^,^^8^ and alterations of pulsatile flow patterns^9^ during ECMO also play a role in altered cerebral autoregulation. Thus, both pre- and intra-ECMO factors can result in hemorrhagic and ischemic complications commonly seen in ECMO. A reliable methodology that can assess the status of cerebral autoregulation during ECMO and provide early indication of neurological injury is critical for optimization of bedside management to improve clinical outcomes.

In the past decade, various methods have been developed to assess the status of cerebral autoregulation based on the relationship between spontaneous fluctuations in blood pressure and CBF surrogates.^10^ Methods in time domain include the calculation of linear correlation coefficient in a moving time window.^11^ The size of the time window was usually fixed, which limits its capacity to assess cerebral autoregulation over multiple time scales.^12^ Methods in frequency domain have used transfer function and magnitude-squared coherence function to describe the frequency-dependent characteristics of cerebral autoregulation.^13^^,^^14^ However, these methods are based on the assumption that changes in blood pressure and CBF are stationary and linearly correlated, which may not hold true, especially in critically ill patients. In contrast, wavelet transform coherence (WTC) is a time-frequency domain analysis that characterizes the cross correlation and relative phase between two signals without *a priori* assumptions of linearity and stationarity.^15^ Our previous work has demonstrated that WTC is a suitable tool to study the dynamic cerebral autoregulation in newborns with hypoxic–ischemic encephalopathy (HIE).^16^

In this study, we implemented WTC to assess the degree of cerebral autoregulation impairment in neonatal and pediatric ECMO and evaluated its usefulness as an early predictor of acute neurological complications. Further, we examined type and duration of ECMO, blood gas changes, and anticoagulation parameters as potential causes of autoregulation impairment.

## 2 Materials and Methods

### 2.1 Participants

This study included patients 0 to 15 years of age who were placed on ECMO from January 2014 to May 2016 at Children’s Health, Dallas. The reasons for ECMO intervention were persistent pulmonary hypertension of the newborn (PPHN), septic shock and/or acute respiratory distress syndrome (ARDS). Patients with pre-existing neurological injuries, underlying congenital heart disease, and CPR patients were excluded due to their predisposition to pre-ECMO cerebral injuries.^17^^–^^19^ All the patients were placed on Rotaflow centrifugal pumps. Cannulation for venoarterial (VA) ECMO was through the carotid artery and internal jugular vein. Cannulation for venovenous (VV) ECMO was through the double lumen venous catheters placed in the right internal jugular vein. Most of the patients were sedated with fentanyl and versed. Dexmedetomidine was used as an adjunct in some patients. Heparin was the anticoagulation drug of choice. The loading dose at the time of cannulation was 50 to 100  units/kg. Heparin infusion was titrated for anticoagulation goals. Bedside activated clotting time in seconds, partial thromboplastin time (PTT) in seconds, and unfractionated heparin (UH) levels (international units/ml) were the measurement parameters. The study was approved by the institutional review board at the University of Texas Southwestern Medical Center, Dallas, and informed consent was waived.

### 2.2 Arterial Blood Gas and Anticoagulation

Measurements of arterial blood gas were obtained from 24-h pre-ECMO to the first 24 h on ECMO as the biggest change occurred during this time period. This included pH, partial pressure of oxygen (${\mathrm{PaO}}_{2}$), and partial pressure of carbon dioxide (${\mathrm{PCO}}_{2}$). Similarly, the minimum, maximum, and mean values of the coagulation indices (PTT and UH) throughout the ECMO run were also calculated.

### 2.3 Autoregulation Monitoring

Mean arterial pressure (MAP) was continuously measured from an indwelling arterial catheter. As a surrogate for CBF, cerebral tissue oxygen saturation ($S_{\mathrm{ct}}O_{2}$) was measured on the forehead using a cerebral oximeter (INVOS^™^ 4100-5100, Somanetics, Troy, Michigan). Both signals were sampled minute-by-minute and recorded throughout the ECMO run except during patient transportation for imaging or other procedures. The recorded data were inspected for spike-like artifacts, which were removed by linear interpolation between neighboring data points.^12^ Then, a second-order polynomial detrending was applied to remove the slow drifts in each signal.

### 2.4 Wavelet Transform Coherence

A detailed description of WTC in studying dynamic cerebral autoregulation is outlined in our previous publication.^16^ Briefly, it characterizes the squared cross-wavelet coherence and relative phase between two paired signals in time-frequency domain.^15^ For a time series x(n) of length N, which is sampled from a continuous signal at a time step of Δt, the continuous wavelet transform is defined as

$$W^{X}(n,s)=\sqrt{\frac{\Delta t}{s}}\overset{N}{\underset{n^{\prime }=1}{\sum }}x(n)\psi_{0}^{*}[(n^{\prime }-n)(\frac{\Delta t}{s})],$$ (1)

where n is a time index, s denotes the wavelet scale that is in inverse proportion to Fourier frequency (s∝1/f), and ^*^ indicates the complex conjugate.

In analogy to Fourier analysis, the cross-wavelet transform of two time series, x(n) and y(n), is defined as

$$W^{XY}(n,s)=W^{X}(n,s)W^{Y^{*}}(n,s),$$ (2)

where the modulus $|W^{XY}(n,s)|$ represents the amount of joint power between x(n) and y(n), and the complex argument $\Delta \varphi (n,s)=\tan^{-1}\{\frac{\text{Im}[W^{XY}(n,s)]}{\text{Re}[W^{XY}(n,s)]}\}$ represents the relative phase between x(n) and y(n).

The squared cross-wavelet coherence, $R^{2}(n,s)$, is defined as

$$R^{2}(n,s)=\frac{{\left|S[S^{-1}W^{XY}(n,s)]\right|}^{2}}{S[S^{-1}{\left|W^{X}(n,s)\right|}^{2}]\cdot S[S^{-1}{\left|W^{Y}(n,s)\right|}^{2}]},$$ (3)

where S is a smoothing operator in the time-frequency domain, which is necessary to remove singularities embedded in the power distributions of x(n) and y(n). $R^{2}(n,s)$ ranges between 0 and 1 and can be conceptualized as a localized correlation coefficient between x(n) and y(n) in the time-frequency domain.

The statistical significance of $R^{2}(n,s)$ between the two paired signals, which were the spontaneous MAP and $S_{\mathrm{ct}}O_{2}$ fluctuations in this study, against background noise can be assessed using the Monte Carlo method.^15^ Briefly, this method generates a large ensemble of surrogate data pairs (n=300) that have the same coefficients as the actual input signals based on the first-order autoregressive (AR1) model. Wavelet coherence is calculated for all of the surrogate data pairs. Then, the significance level of $R^{2}(n,s)$ of the actual input signals is determined by comparing with those from the surrogate data pairs at each time and wavelet scale. Here, a 95% confidence interval (p<0.05) is used for statistical testing. Based on our observation, this confidence interval corresponded to a critical value of $R^{2}(n,s)$ narrowly between 0.71 and 0.73 across all the patients.

The relative phase between the two paired signals, defined as $\Delta \varphi (n,s)=\tan^{-1}\{\frac{\text{Im}[W^{XY}(n,s)]}{\text{Re}[W^{XY}(n,s)]}\}$, ranges between −π and π. In the previous publication,^16^ we characterized the phase relationship in four separate ranges: Δϕ=0±π/4, π/2±π/4, π±π/4, and −π/2±π/4. Each phase range represents a distinct pattern of coherence and might be related to different underlying physiological mechanisms: Δϕ=0±π/4 represents an in-phase pattern where the two signals oscillate in the same directions, Δϕ=π±π/4 represents an antiphase pattern where the two signals oscillate in opposite directions, and both Δϕ=π/2±π/4 and −π/2±π/4 represent an asynchronous pattern where the two signals have significant phase differences.

In this study, we focused on a pressure-passive state of cerebral autoregulation,^11^^,^^20^^,^^21^ i.e., the patient’s changes in blood pressure cause simultaneous changes in cerebral oxygenation in the same directions. The pressure-passive state is a vital sign of an impaired autoregulation system^21^ and results in significant in-phase coherence between the MAP and $S_{\mathrm{ct}}O_{2}$ signals.^16^ Thus, a phase range of Δϕ∈0±π/4 was selected as the range of interest. Within this phase range, the percentage of significant coherence, P(s), was quantified as the percentage of time during which the $\mathrm{MAP}\to S_{\mathrm{ct}}O_{2}$ coherence was statistically significant from the background noise (p<0.05) via the Monte Carlo method.^16^ P(s) was a function of wavelet scale s (i.e., a function of 1/f) and represented the scale/frequency characteristics of $\mathrm{MAP}\to S_{\mathrm{ct}}O_{2}$ coherence. Cerebral autoregulation is widely recognized as a frequency-dependent phenomenon.^13^^,^^14^ Previous studies have shown that the $\mathrm{MAP}\to S_{\mathrm{ct}}O_{2}$ coherence with a scale range of ≤30  min was most relevant to the pressure-passive state.^11^^,^^20^^,^^21^ In this study, we calculated the mean value of P(s), $P_{\text{mean}}$, in a scale range of 8 to 32 min as an index of cerebral autoregulation impairment.^16^

### 2.5 Neuroimaging Assessment and Scoring

For all neonates and children with open fontanels, head ultrasound was done every day for the first three days of their ECMO run and more frequently if clinically indicated. If there was any ongoing concern of their neurological status, an emergent computed tomography (CT) scan was conducted while on ECMO, which consisted of 3-mm contiguous axial images from C1 to the cranial vertex (Somatom, Siemens, Erlangen, Germany). Post-ECMO magnetic resonance imaging (MRI) at 1.5 or 3 T was done on all the patients prior to their discharge (Achieva and Intera, Philips Healthcare, Best, the Netherlands). The standard protocol consisted of T1-weighted sagittal, T2-weighted or fluid-attenuated inversion recovery axial images, T2-weighted coronal, and diffusion-weighted axial images. Some patients also had susceptibility-weighted axial images. Neonates had T1-weighted axial imaging.

Neurological abnormalities on brain imaging were evaluated by a neuroradiologist (M.C.M), who was blinded to the patients’ clinical conditions and autoregulation measurements, based on a scoring system that had been previously validated among ECMO patients.^22^^,^^23^ It included three categories of abnormalities: bleeding, parenchymal lesions (ischemia or infarction), and ventricular dilatation. Each category was assigned a score ranging from 1 to 3 and then multiplied by a relative weight factor and added to the total score. In case a patient had both CT imaging during ECMO and MRI post-ECMO, the CT imaging was analyzed for scoring purposes. Neuroimaging scores were grouped into the following three categories: normal (score=0), mild to moderate (score=0.5 to 6.0), and severe (score=6.0 to 18.0).^23^

### 2.6 Statistical Analysis

Both univariate and multivariate linear regression analyses were conducted to investigate: (1) the relationship between the individual cerebral autoregulation indices and neuroimaging scores, which were the primary outcomes in this study and (2) the effects of ECMO type (VV and VA) and duration, blood gas changes (pH, ${\mathrm{PaO}}_{2}$, and ${\mathrm{PCO}}_{2}$), and coagulation indices (PTT and UH) on the individual autoregulation indices and neuroimaging scores. These analyses were conducted in two separate age groups (neonates and children) as well as in the entire cohort.

## 3 Results

### 3.1 Patient Characteristics

Twenty-nine pediatric patients who met the inclusion criteria were enrolled during the study period. Four were not included for data analysis due to loss of MAP data (two) or $S_{\mathrm{ct}}O_{2}$ data (two). The final sample size was 25 (8 males and 17 females). Among these patients, 9 had VV ECMO, 12 had VA ECMO, and the other 4 initially had VV ECMO and then were converted to VA ECMO. Neonates (≤4  weeks of age) were the biggest subpopulation and had relatively unifying diagnosis (10 out of 11 were placed on ECMO for PPHN). Children had more variable diagnoses. Individual characteristics, primary and secondary diagnoses, ECMO type and duration, and patient survival to hospital discharge are detailed in Table 1.

**Table 1:** Characteristics, primary and secondary diagnoses, type and duration of ECMO, and survival status (yes or no) of the neonatal patients.

	Patient No.	Age	Gender	Diagnosis		ECMO		Survival
				Primary	Secondary	Type	Duration (h)	
Neonates	1	<1 week	F	PPHN	CDH	VA	545	No
	3	<1 week	F	PPHN	Meconium aspiration	VA	102	Yes
	4	<1 week	F	PPHN	Meconium aspiration	VA	62	No
	6	<1 week	M	PPHN	Meconium aspiration	VV	75	Yes
	12	<1 week	F	ARDS and septic shock	Adenovirus	VV→VA	263	No
	13	<1 week	F	PPHN	Meconium aspiration	VA	89	Yes
	14	<1 week	M	PPHN	Neonatal lung disease	VA	112	Yes
	17	<1 week	M	PPHN	Septic shock	VA	56	Yes
	19	<1 week	M	PPHN	Meconium aspiration	VA	58	Yes
	21	<1 week	F	PPHN	Meconium aspiration	VA	106	Yes
	23	<1 week	F	PPHN	Meconium aspiration	VV	80	Yes
Children	2	3 months	F	ARDS	Rhinovirus infection	VV	227	No
	5	8 years	F	ARDS	Pulmonary contusion secondary to trauma	VV	140	Yes
	7	7 months	M	ARDS	Influenza A	VV	346	Yes
	8	10 months	F	Septic shock	HLH and influenza	VV→VA	310	No
	9	2 years	F	ARDS	RSV and *Moraxella* pneumonia	VV→VA	938	Yes
	10	14 years	F	Septic shock	*Neisseria meningitidis*	VA	48	No
	11	15 years	M	ARDS	Fluid overload and sepsis	VV	368	Yes
	15	14 years	F	ARDS	ALL and BMT	VV	144	Yes
	16	3 months	M	ARDS	Parainfluenza and *Haemophilus influenzae*	VV→VA	330	Yes
	18	7 years	M	Septic shock	*Streptococcus pneumonia*	VA	153	Yes
	20	6 weeks	F	ARDS	Influenza, RSV, *Moraxella*	VV	396	No
	22	4 months	F	ARDS	Septic shock and liver failure	VA	71	No
	24	2 years	F	ARDS	RSV and ALL	VV	550	Yes
	25	6 years	F	Septic shock	ALL	VA	105	Yes

Note: ALL = acute lymphatic leukemia, ARDS = acute respiratory distress syndrome, BMT = bone marrow transplant, CDH = congenital diaphragmatic hernia, HLH = hemophagocytic lymphohistiocytosis, PPHN = persistent pulmonary hypertension of the newborn, RSV = respiratory syncytial virus, VA = venoarterial, and VV = venovenous.

Table 2 shows the neuroimaging modality, timing, and score for each individual patient. In all but one patient, MRI was done within a week of coming off ECMO support. Per this table, 8 patients (32%) had normal neuroimaging, 7 (28%) had mild to moderate neuroimaging abnormalities, and the other 10 (40%) had severe neuroimaging abnormalities. The ECMO type (VA and VV) and duration did not show significant effect on neuroimaging scores.

**Table 2:** Intra-ECMO autoregulation indices and neuroimaging scores of the patients.

	Patient No.	Autoregulation index (%)	Neuroimaging		
			Modality	Time	Total score
Neonates	1	1.7	Ultrasound	During ECMO	0
	3	0.8	MRI	3 days post-ECMO	0
	4	8.7	MRI	4 days post-ECMO	10.5
	6	2.0	MRI	2 days post-ECMO	0
	12	12.0	CT	During ECMO	21
	13	4.0	MRI	2 days post-ECMO	0
	14	3.5	Ultrasound	During ECMO	1
	17	2.4	MRI	2 days post-ECMO	11
	19	1.1	MRI	4 days post-ECMO	0
	21	0.6	CT	During ECMO	0
	23	1.2	MRI	23 days post-ECMO	1
Children	2	3.1	CT	During ECMO	1
	5	11.9	CT	During ECMO	10
	7	2.5	MRI	2 days post-ECMO	4
	8	1.1	CT	During ECMO	11
	9	4.1	MRI	5 days post-ECMO	5
	10	15.3	CT	During ECMO	16.5
	11	8.0	CT	During ECMO	6
	15	1.8	MRI	7 days post-ECMO	9
	16	1.1	CT	During ECMO	4
	18	14.4	MRI	5 days post-ECMO	9
	20	4.5	Ultrasound	During ECMO	0
	22	8.4	CT	During ECMO	9
	24	1.0	MRI	5 days post-ECMO	8
	25	4.0	MRI	4 days post-ECMO	0

### 3.2 Autoregulation Impairment During Extracorporeal Membrane Oxygenation

The spontaneous fluctuations of MAP and $S_{\mathrm{ct}}O_{2}$ during ECMO were mostly incoherent among patients who had normal neuroimaging scores. In contrast, patients with significant in-phase $\mathrm{MAP}\to S_{\mathrm{ct}}O_{2}$ coherence always had abnormal neuroimaging scores. Additionally, one neonate and two children had abnormal neuroimaging scores but low in-phase $\mathrm{MAP}\to S_{\mathrm{ct}}O_{2}$ coherence. The neonate (patient 17) had PPHN secondary to group B Streptococcus (GBS) infection with concern for possible central nervous system GBS infection, which could have predisposed the infant to abnormal neuroimaging. The two children (patients 8 and 15) had leukemia with significant brain volume loss from their underlying immunocompromised state.

**Fig. 1:**
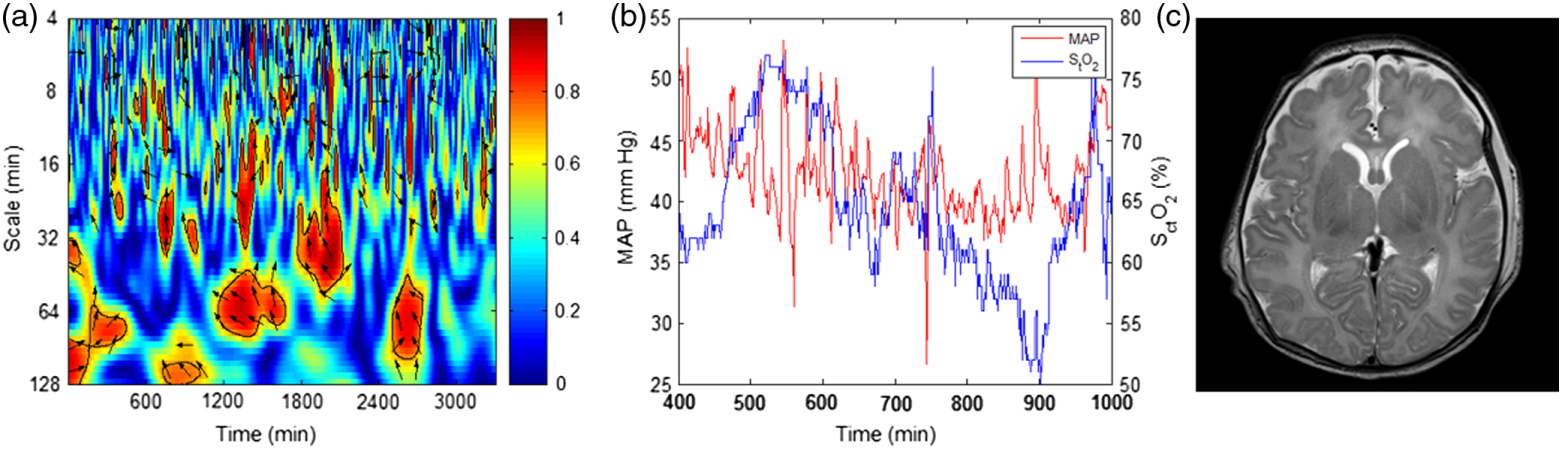
Autoregulation and neuroimaging results from a patient (patient 6: neonatal male) who was placed on VV ECMO for meconium aspiration secondary to PPHN. (a) Partial enlarged figure of WTC between the spontaneous fluctuations of MAP and $S_{\mathrm{ct}}O_{2}$. In this graph, the x-axis represents the time, the y-axis represents the wavelet scale (in inverse proportion to Fourier frequency), the color scale represents the squared cross-wavelet coherence ($R^{2}$) that ranges from 0 to 1, and the black line contours designate the areas of significant coherence (p<0.05) identified through Monte Carlo simulation. The arrows designate the relative phase between MAP and $S_{\mathrm{ct}}O_{2}$: a rightward-pointing arrow indicates in-phase coherence and leftward-pointing arrow indicates antiphase coherence. (b) A segment of real-time MAP and $S_{\mathrm{ct}}O_{2}$ data. (c) MRI brain image acquired 2 days after ECMO.

**Fig. 2:**
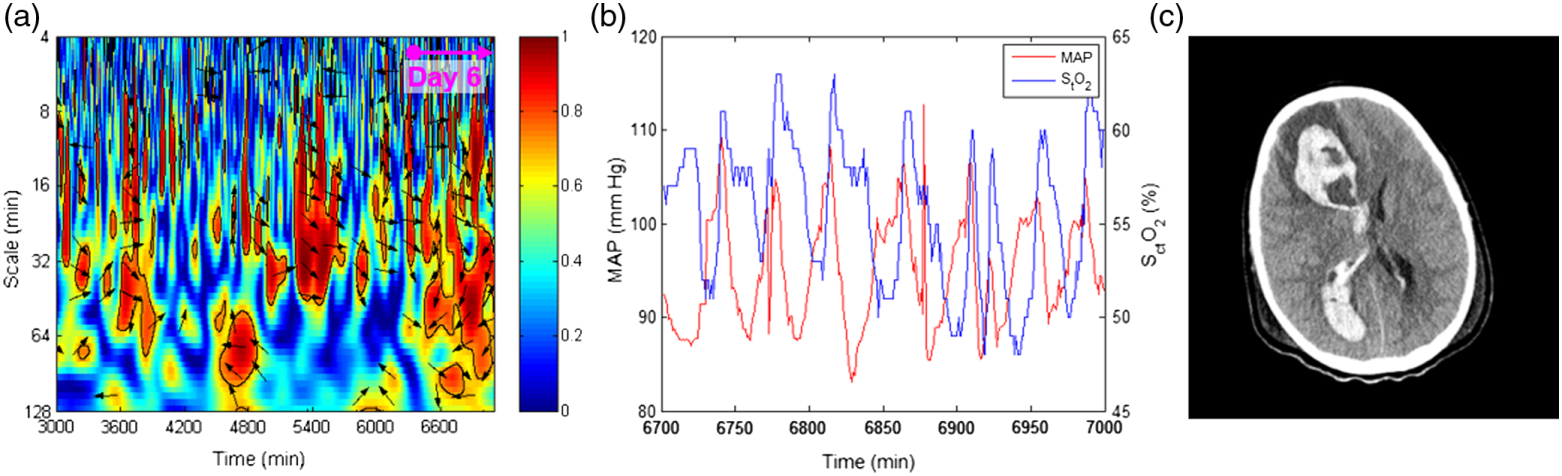
Autoregulation and neuroimaging results from a patient (patient 5: 8-year-old female) who was placed on VV ECMO for pulmonary contusion secondary to ARDS s/p motor vehicle collision. (a) Partial enlarged figures of WTC between the spontaneous fluctuations of MAP and $S_{\mathrm{ct}}O_{2}$. In this graph, the x-axis represents the time, the y-axis represents the wavelet scale (in inverse proportion to Fourier frequency), the color scale represents the squared cross-wavelet coherence ($R^{2}$) that ranges from 0 to 1, and the black line contours designate the areas of significant coherence (p<0.05) identified through Monte Carlo simulation. The arrows designate the relative phase between MAP and $S_{\mathrm{ct}}O_{2}$: a rightward-pointing arrow indicates in-phase coherence and leftward-pointing arrow indicates antiphase coherence. (b) A segment of real-time MAP and $S_{\mathrm{ct}}O_{2}$ data. (c) CT brain image acquired during ECMO.

For instance, results from two representative patients are shown in Figs. 1 and 2 respectively, and described in detail below.

Patient 6 was a neonatal male who was placed on VV ECMO for meconium aspiration secondary to PPHN. Incoherent MAP and $S_{\mathrm{ct}}O_{2}$ changes were seen during the majority of the time of ECMO run. This was confirmed in a segment of real-time MAP and $S_{\mathrm{ct}}O_{2}$ data. The patient survived, and his post-ECMO MRI was normal.

Patient 5 was an 8-year-old female who was placed on VV ECMO for pulmonary contusion secondary to ARDS s/p motor vehicle collision. Her initial head CT on admission had no evidence of intracranial bleeding. She was found to have a large intracranial bleeding on day 6 and was emergently weaned off from ECMO and decannulated. Intermittent in-phase coherence between the MAP and $S_{\mathrm{ct}}O_{2}$ changes was seen in a scale range of 8 to 64 min throughout her ECMO run. It became much more apparent on day 6, ∼10  h prior to decannulation. Her CT image after decannulation showed extensive intraventricular and right frontal parenchymal hemorrhage with obstructive hydrocephalus.

**Fig. 3:**
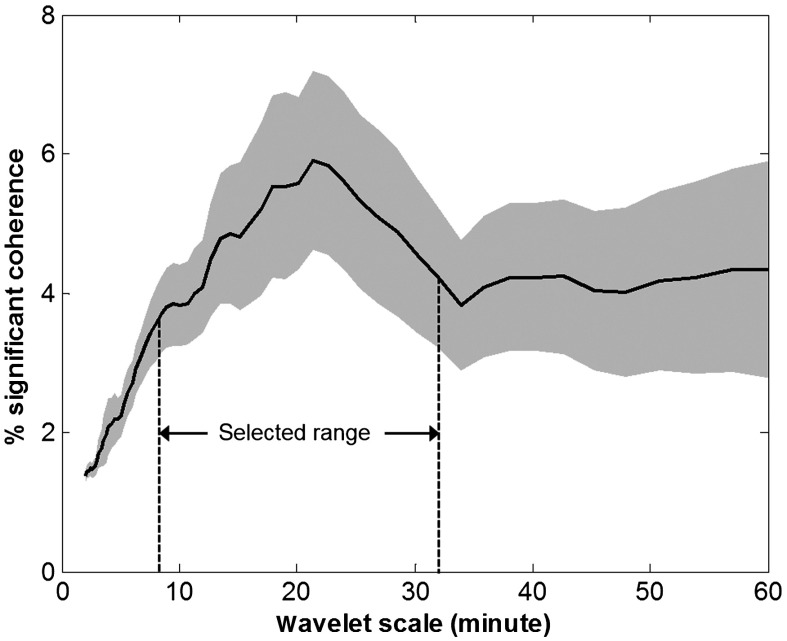
Percentage of significant coherence, P(s), derived from the in-phase $\mathrm{MAP}\to S_{\mathrm{ct}}O_{2}$ coherence (i.e., Δφ∈0±π/4). In this graph, the x-axis represents the wavelet scale, s, which is in inverse proportion to Fourier frequency. The y-axis represents the percentage of time during which the $\mathrm{MAP}\to S_{\mathrm{ct}}O_{2}$ coherence was statistically significant over the background noise (p<0.05). Therefore, P(s) represented the scale/frequency characteristics of the $\mathrm{MAP}\to S_{\mathrm{ct}}O_{2}$ coherence. For ECMO patients, predominant in-phase $\mathrm{MAP}\to S_{\mathrm{ct}}O_{2}$ coherence was seen in a wavelet scale range of 8 to 32 min (the shaded area), which corresponded to a frequency range of 0.0005 to 0.002 Hz. This range was selected to calculate the autoregulation index.

At the group level, the most predominant in-phase $\mathrm{MAP}\to S_{\mathrm{ct}}O_{2}$ coherence was seen in a scale range of 8 to 32 min (Fig. 3), which was consistent with previous findings on pressure-passive autoregulation.^11^^,^^20^^,^^21^ Therefore, the mean percentage of significant in-phase $\mathrm{MAP}\to S_{\mathrm{ct}}O_{2}$ coherence was calculated in this scale range as an index of intra-ECMO autoregulation impairment.

### 3.3 Autoregulation Index versus Neuroimaging Score

**Fig. 4:**
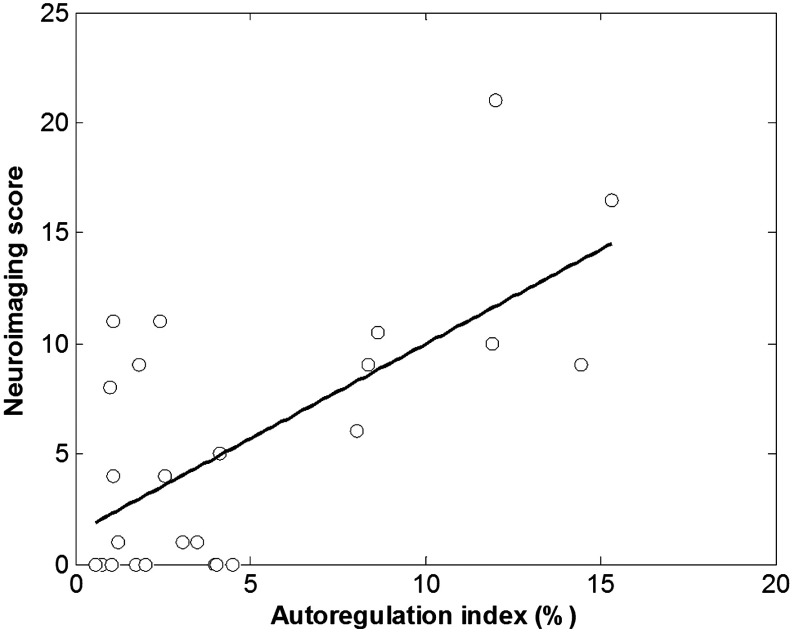
Correlation between autoregulation index and neuroimaging score.

As shown in Fig. 4, significant correlation between the autoregulation indices and neuroimaging scores was found over the entire cohort (R=0.66; p<0.0001). Thus, high degrees of cerebral autoregulation impairment during ECMO were indicative of severe neuroimaging abnormalities.

### 3.4 Effects of Arterial Blood Gas and Anticoagulation Parameters on Cerebral Autoregulation

Since the biggest changes in pH, ${\mathrm{PaO}}_{2}$, and ${\mathrm{PCO}}_{2}$ generally occurred from pre-ECMO to first 24 h of ECMO (Table 3), we analyzed the effects of these changes on cerebral autoregulation impairment. Specifically, the individual pH and ${\mathrm{PaO}}_{2}$ changes were calculated from the minimum values in the 24-h pre-ECMO to the maximum values in the first 24 hours of ECMO, which showed no significant correlation with autoregulation index. Similarly, the individual ${\mathrm{PCO}}_{2}$ change was calculated from the maximum value in the 24-h pre-ECMO to the minimum value in the first 24 h of ECMO, which showed no significant correlation with autoregulation index either.

**Table 3:** Arterial blood gas changes from 24-h pre-ECMO to the first 24 h of ECMO.

	Patient No.	pH	${\mathrm{PaO}}_{2}$ (mm Hg)	${\mathrm{PCO}}_{2}$ (mm Hg)
Neonates	1	0.46	156	−74
	3	0.22	114	2
	4	0.41	55	−39
	6	0.21	13	−19
	12	0.21	205	−32
	13	0.09	97	−10
	14	0.17	56	19
	17	0.19	140	−14
	19	0.27	55	−20
	21	0.40	95	−12
	23	0.34	52	−46
Children	2	0.22	13	−41
	5	0.54	8	−60
	7	0.32	23	−40
	8	0.39	189	−94
	9	0.21	76	−26
	10	0.26	175	−9
	11	0.21	23	−31
	15	0.25	1	−48
	16	0.42	18	−51
	18	0.40	117	−12
	20	0.26	106	−38
	22	0.19	287	−14
	24	0.29	−1	−54
	25	0.15	251	−2

Finally, the anticoagulation parameters (PTT and UH) (Table 4) were not significantly associated with the patients’ neuroimaging scores.

**Table 4:** Anticoagulation parameters throughout ECMO run.

	Patient No.	PTT (s)			UH (international units/ml)			International normalized ratio		
		Min	Mean	Max	Min	Mean	Max	Min	Mean	Max
Neonates	1	21.7	149.1	200	0.10	0.29	0.94	0.8	1.2	1.9
	3	78	138.9	200	0.16	0.37	0.60	1	1.1	1.2
	4	71.8	101	200	0.10	0.13	0.23	1.4	1.5	1.6
	6	77	146.3	200	0.10	0.28	0.39	1.2	1.4	1.6
	12	33.1	115.1	200	0.10	0.16	0.34	0.9	1.1	1.8
	13	93.8	140.9	172.9	0.24	0.37	0.53	0.9	1.3	1.7
	14	830	138.3	200	0.12	0.48	1.01	1.1	1.2	1.5
	17	72.5	126.4	200	0.10	0.33	1.10	1.4	1.7	2.2
	19	37.8	108.3	200	0.10	0.14	0.30	1.4	2	3.6
	21	74.1	106.4	139.4	0.10	0.14	0.20	1.1	1.6	2.1
	23	136.6	177.7	200	0.11	0.22	0.34	1.5	1.8	2
Children	2	52.9	84.9	111.1	0.10	0.43	0.72	0.9	1	1.3
	5	53.5	88.7	200	0.15	0.33	0.70	1	1.1	1.2
	7	52.5	85	200	0.17	0.36	0.71	0.9	1.1	1.2
	8	49.1	74.7	158.3	0.10	0.37	0.94	0.8	1.3	2.2
	9	35.9	52.3	131.3	0.10	0.29	0.59	0.8	1	1.2
	10	200	200	200	0.10	0.10	0.10	2.2	2.8	3.6
	11	38	51.8	70.7	0.10	0.13	0.31	1	1.2	1.6
	15	54.9	85.9	200	0.24	0.31	0.36	1	1.1	1.3
	16	56.7	102.1	154.9	0.10	0.27	0.48	1	1.1	1.5
	18	58.1	102.7	200	0.10	0.30	0.52	1	1.4	2.6
	20	35.4	92.9	200	0.10	0.21	0.56	1	1.3	2.7
	22	85.3	130	200	0.12	0.18	0.23	1.5	1.6	1.7
	24	37.8	70	131.5	0.10	0.14	0.20	0.9	1	1.2
	25	57	66.3	78	0.11	0.19	0.27	1.1	1.4	1.8

## 4 Discussion

ECMO is a rapidly advancing technology and has claimed an important role in the management algorithms for disease states, such as ARDS and septic shock, in the pediatric intensive care unit. In this study, we continuously monitored cerebral autoregulation throughout the course of ECMO therapy. We found intra-ECMO autoregulation impairment was apparent even before clinically observable changes occur at the bedside (such as the patient 5 as shown in Fig. 2). Furthermore, the degrees of cerebral autoregulation impairment derived from WTC correlated with the patients’ neuroimaging abnormalities. This finding remained constant for both VA ECMO and VV ECMO in contrast to evidence of increased incidence of neurological complication with carotid artery cannulation in the literature.^24^

Currently, there are no reliable clinical methods at the bedside to evaluate subclinical neurologic events during ECMO. While transcranial Doppler ultrasound may be performed in any patient, grayscale ultrasound imaging with evaluation of the brain parenchyma can be accomplished only in neonates with an open fontanelle for an acoustic window to visualize the brain.^25^ This leaves diagnostic imaging such as CT scan^26^ as an available option, which is also challenging because transportation of these patients is labor-intensive and associated with risks.

Cerebral autoregulation impairment during ECMO has been previously described in animal models,^9^^,^^27^^–^^29^ which raised concerns that it might be a major contributor to neurological injuries. A recent study on human infants assessed cerebral autoregulation via sequentially altering ECMO flow.^30^ However, this study was limited by the fact that the assessment was done for only an hour while ECMO therapy could last for days to weeks with neurological events occurring anytime during this course. It would be extremely difficult to perform the same assessment repeatedly and safely. Therefore, in this study, we utilized the spontaneous fluctuations of MAP and $S_{\mathrm{ct}}O_{2}$ to assess the dynamic status of cerebral autoregulation. We further implemented WTC to overcome the nonlinearity and nonstationarity of the spontaneous signals and to derive a quantitative index of cerebral autoregulation impairment. We provided initial evidence that such an index was indicative of the patients’ neuroimaging abnormalities. Since the spontaneous fluctuations of MAP and $S_{\mathrm{ct}}O_{2}$ can be recorded continuously in clinical settings, such an approach has the potential to provide real-time feedbacks on the patients’ neurological conditions at the bedside.

In the next step, we examined the arterial blood gas and anticoagulation parameters as potential causes of impaired autoregulation in ECMO. Rapid changes in pH and ${\mathrm{PCO}}_{2}$ can have significant effects on CBF.^31^^,^^32^ In a recent retrospective study on adult patients supported on VV ECMO, patients who developed intracranial bleeding were found to have a rapid decrease in ${\mathrm{PCO}}_{2}$ at the initiation of ECMO.^33^ However, that was not the case in our cohort of patients. This could be due to our center practice of changing pH and ${\mathrm{PCO}}_{2}$ gradually once ECMO was initiated. Moreover, we did not find any significant effect of heparin dose on cerebral autoregulation, which is in line with previous studies on neonates with intracranial complications.^34^^,^^35^

There are several limitations in this study. First, this was a pilot study conducted at a single ECMO center. The sample size was small, which limited our capability to address the differences among patients with different subtypes of diagnoses. The results from this study should be interpreted with caution. Second, $S_{\mathrm{ct}}O_{2}$ has been widely used as a surrogate for CBF in previous human studies^11^^,^^36^ as well as in this study, which can be recorded conveniently from a standard cerebral oximeter. The validity of this variable has been demonstrated in animal models under hypotension,^37^ cardiac arrest, and hypothermia.^38^^,^^39^ However, it remains an indirect measure of CBF and could be influenced by other cerebrovascular factors. Diffuse correlation spectroscopy (DCS)^40^ is an emerging technology that can measure regional CBF directly. The device is compact and suitable for bedside monitoring. Future studies should consider using DCS in supplement to the traditional cerebral oximetry. Last, we have relied on routine neuroimaging assessments to score brain injuries in the patients. It involved utilization of multiple imaging modalities during and/or after ECMO run, which might have increased variability in the patients’ neuroimaging scores. In general, MRI is considered as the gold standard for detection of ischemic injury, offering techniques such as diffusion-weighted imaging. However, MRI cannot be performed while patients are on ECMO. Head ultrasound and CT can be performed during ECMO, but both are less sensitive to ischemic lesions. On the other hand, lesions, such as significant hemorrhage, hydrocephalus, large infarctions, or atrophy, would not be difficult to detect by high-quality ultrasound or CT. Large and neurologically significant lesions were the primary objects of assessment in this study. Therefore, we believe that the utilization of multiple imaging modalities did not significantly affect the neuroimaging scores in this study.

## References

[1] Lequier L., “Extracorporeal life support in pediatric and neonatal critical care: a review,” J. Intensive Care Med. 19, 243–258 (2004). 10.1177/0885066604267650 15358943

[2] “ECLS registry report: international summary July 2016,” http://www.elsonet.org/index.php/ registry/statistics/limited.html (1 8 2016).

[3] UK Collaborative ECMO Trial Group, “UK collaborative randomised trial of neonatal extracorporeal membrane oxygenation,” Lancet 348, 75–82 (1996). LANCAO 0140-6736 10.1016/S0140-6736(96)04100-1 8676720

[4] Polito A. et al., “Neurologic complications in neonates supported with extracorporeal membrane oxygenation. An analysis of ELSO registry data,” Intensive Care Med. 39(9), 1594–1601 (2013). ICMED9 0342-4642 10.1007/s00134-013-2985-x 23749154

[5] Paulson O. B. Strandgaard S. Edvinsson L., “Cerebral autoregulation,” Cerebrovasc. Brain Metab. Rev. 2(2), 161–192 (1990). 2201348

[6] Short B. L., “The effect of extracorporeal life support on the brain: a focus on ECMO,” Semin. Perinatol. 29(1), 45–50 (2005). SEMPDU 0146-0005 10.1053/j.semperi.2005.02.007 15921152

[7] Liem K. D. et al., “Cerebral oxygenation and hemodynamics during induction of extracorporeal membrane oxygenation as investigated by near infrared spectrophotometry,” Pediatrics 95(4), 555–561 (1995). PEDIAU 0031-4005 7700758

[8] Fenik J. C. Rais-Bahrami K., “Neonatal cerebral oximetry monitoring during ECMO cannulation,” J. Perinatol. 29(5), 376–381 (2009). JOPEEIJOPEEI 0743-8346 10.1038/jp.2008.231 19158806

[9] Short B. L. et al., “Impairment of cerebral autoregulation during extracorporeal membrane oxygenation in newborn lambs,” Pediatr. Res. 33(3), 289–294 (1993). PEREBL 0031-3998 10.1203/00006450-199303000-00018 8460067

[10] Panerai R. B., “Assessment of cerebral pressure autoregulation in humans—a review of measurement methods,” Physiol. Meas. 19, 305–338 (1998). PMEAE3 0967-3334 10.1088/0967-3334/19/3/001 9735883

[11] Gilmore M. M. et al., “Relationship between cerebrovascular dysautoregulation and arterial blood pressure in the premature infant,” J. Perinatol. 31, 722–729 (2011). JOPEEI 0743-8346 10.1038/jp.2011.17 21372795

[12] Chalak L. F. et al., “Cerebral hemodynamics in asphyxiated newborns undergoing hypothermia therapy: pilot findings using a multiple-time-scale analysis,” Pediatr. Neurol. 55, 30–36 (2016). 10.1016/j.pediatrneurol.2015.11.010 26858217 PMC4748172

[13] Zhang R. et al., “Transfer function analysis of dynamic cerebral autoregulation in humans,” Am. J. Physiol. 274, H233–H241 (1998). AJPHAP 0002-9513 9458872 10.1152/ajpheart.1998.274.1.h233

[14] Zhang R. et al., “Autonomic neural control of dynamic cerebral autoregulation in humans,” Circulation 106, 1814–1820 (2002). CIRCAZ 0009-7322 10.1161/01.CIR.0000031798.07790.FE 12356635

[15] Grinsted A. Moore J. C. Jevrejeva S., “Application of the cross wavelet transform and wavelet coherence to geophysical time series,” Nonlinear Processes Geophys. 11(5–6), 561–566 (2004).1607-7946 10.5194/npg-11-561-2004

[16] Tian F. et al., “Wavelet coherence analysis of dynamic cerebral autoregulation in neonatal hypoxic–ischemic encephalopathy,” NeuroImage Clin. 11, 124–132 (2016). 10.1016/j.nicl.2016.01.020 26937380 PMC4753811

[17] Donofrio M. T. Massaro A. N., “Impact of congenital heart disease on brain development and neurodevelopmental outcome,” Int. J. Pediatr. 2010, 359390 (2010). 10.1155/2010/359390 20862365 PMC2938447

[r18] Miller S. P. et al., “Abnormal brain development in newborns with congenital heart disease,” N. Engl. J. Med. 357(19), 1928–1938 (2007). NEJMAG 0028-4793 10.1056/NEJMoa067393 17989385

[19] McQuillen P. S. et al., “Temporal and anatomic risk profile of brain injury with neonatal repair of congenital heart defects,” Stroke 38(2 Suppl.), 736–741 (2007). 10.1161/01.STR.0000247941.41234.90 17261728

[20] Tsuji M. et al., “Cerebral intravascular oxygenation correlates with mean arterial pressure in critically ill premature infants,” Pediatrics 106, 625–632 (2000). PEDIAU 0031-4005 10.1542/peds.106.4.625 11015501

[21] Soul J. S. et al., “Fluctuating pressure-passivity is common in the cerebral circulation of sick premature infants,” Pediatr. Res. 61, 467–473 (2007). PEREBL 0031-3998 10.1203/pdr.0b013e31803237f6 17515873

[22] Taylor G. A. et al., “Intracranial abnormalities in infants treated with extracorporeal membrane oxygenation: imaging with US and CT,” Radiology 165(3), 675–678 (1987). RADLAX 0033-8419 10.1148/radiology.165.3.3317499 3317499

[23] Bulas D. I. et al., “Neonates treated with ECMO: predictive value of early CT and US neuroimaging findings on short-term neurodevelopmental outcome,” Radiology 195(2), 407–412 (1995). RADLAX 0033-8419 10.1148/radiology.195.2.7536947 7536947

[24] Teele S. A. et al., “The association of carotid artery cannulation and neurologic injury in pediatric patients supported with venoarterial extracorporeal membrane oxygenation,” Pediatr. Crit. Care Med. 15(4), 355–361 (2014). 10.1097/PCC.0000000000000103 24622166

[25] van Heijst A. F. de Mol A. C. Ijsselstijn H., “ECMO in neonates: neuroimaging findings and outcome,” Semin. Perinatol. 38(2), 104–113 (2014). SEMPDU 0146-0005 10.1053/j.semperi.2013.11.008 24580766

[26] Lidegran M. K. et al., “Cranial CT for diagnosis of intracranial complications in adult and pediatric patients during ECMO: clinical benefits in diagnosis and treatment,” Acad. Radiol. 14(1), 62–71 (2007). 10.1016/j.acra.2006.10.004 17178367

[27] Walker L. K. Short B. L. Traystman R. J., “Impairment of cerebral autoregulation during venovenous extracorporeal membrane oxygenation in the newborn lamb,” Crit. Care Med. 24, 2001–2006 (1996). CCMDC7 0090-3493 10.1097/00003246-199612000-00012 8968268

[r28] Rosenberg A. A. Kinsella J. P., “Effect of extracorporeal membrane oxygenation on cerebral hemodynamics in newborn lambs,” Crit. Care Med. 20, 1575–1581 (1992). CCMDC7 0090-3493 10.1097/00003246-199211000-00016 1424702

[29] Ingyinn M. et al., “Altered cerebrovascular responses after exposure to venoarterial extracorporeal membrane oxygenation: role of the nitric oxide pathway,” Pediatr. Crit. Care Med. 7(4), 368–373 (2006). 10.1097/01.PCC.0000225372.38460.12 16738508

[30] Papademetriou M. D. et al., “Multichannel near infrared spectroscopy indicates regional variations in cerebral autoregulation in infants supported on extracorporeal membrane oxygenation,” J. Biomed. Opt. 17(6), 067008 (2012). JBOPFO 1083-3668 10.1117/1.JBO.17.6.067008 22734786

[31] Lassen N. A. Christensen M. S., “Physiology of cerebral blood flow,” Br. J. Anaesth. 48(8), 719–734 (1976). BJANAD 0007-0912 10.1093/bja/48.8.719 7284

[32] Meng L. Gelb A. W., “Regulation of cerebral autoregulation by carbon dioxide,” Anesthesiology 122, 196–205 (2015). ANESAV 0003-3022 10.1097/ALN.0000000000000506 25401418

[33] Luyt C. E. et al., “Brain injury during venovenous extracorporeal membrane oxygenation,” Intensive Care Med. 42(5), 897–907 (2016). ICMED9 0342-4642 10.1007/s00134-016-4318-3 27007107

[34] Doymaz S. Zinger M. Sweberg T., “Risk factors associated with ICH in neonates with PPHN on ECMO,” J. Intensive Care 3, 6 (2015). 10.1186/s40560-015-0071-x 25705431 PMC4336126

[35] Dela Cruz T. V. et al., “Risk factors for intracranial hemorrhage in the extracorporeal membrane oxygenation patient,” J. Perinatol. 17, 18–23 (1997). JOPEEI 0743-8346 9069059

[36] Caicedo A. et al., “Cerebral tissue oxygenation and regional oxygen saturation can be used to study cerebral autoregulation in prematurely born infants,” Pediatr. Res. 69, 548–553 (2011). PEREBL 0031-3998 10.1203/PDR.0b013e3182176d85 21364491

[37] Brady K. M. et al., “Continuous time-domain analysis of cerebrovascular autoregulation using near-infrared spectroscopy,” Stroke 38, 2818–2825 (2007). SJCCA7 0039-2499 10.1161/STROKEAHA.107.485706 17761921 PMC2377358

[38] Lee J. K. et al., “Cerebral blood flow and cerebrovascular autoregulation in a swine model of pediatric cardiac arrest and hypothermia,” Crit. Care Med. 39, 2337–2345 (2011). CCMDC7 0090-3493 10.1097/CCM.0b013e318223b910 21705904 PMC3178742

[39] Lee J. K. et al., “Noninvasive autoregulation monitoring in a swine model of pediatric cardiac arrest,” Anesth. Analg. 114, 825–836 (2012). 10.1213/ANE.0b013e31824762d5 22314692 PMC3310318

[40] Durduran T. Yodh A. G., “Diffuse correlation spectroscopy for non-invasive, microvascular cerebral blood flow measurement,” NeuroImage 85(Pt. 1), 51–63 (2014). NEIMEF 1053-8119 10.1016/j.neuroimage.2013.06.017 23770408 PMC3991554

